# The Role of Habitat in the Persistence of Fire Ant Populations

**DOI:** 10.1371/journal.pone.0078580

**Published:** 2013-10-21

**Authors:** Walter R. Tschinkel, Joshua R. King

**Affiliations:** 1 Department of Biological Science, Florida State University, Tallahassee, Florida, United States of America; 2 Biology Department, University of Central Florida, Orlando, Florida, United States of America; Field Museum of Natural History, United States of America

## Abstract

The association of the exotic fire ant, *Solenopsis invicta* with man-modified habitats has been amply demonstrated, but the fate of such populations if ecological succession proceeds has rarely been investigated. Resurvey of a fire ant population in a longleaf pine plantation after 25 years showed that the recovery of the site from habitat disturbance was associated with a large fire ant population decline. Most of the persisting colonies were associated with the disturbance caused by vehicle tracks. In a second study, mature monogyne fire ant colonies that had been planted in experimental plots in native groundcover of the north Florida longleaf pine forest had mostly vanished six years later. These observations and experiments show that *S. invicta* colonies rarely persist in the native habitat of these pine forests, probably because they are not replaced when they die. A single site harbored a modest population of polygyne fire ants whose persistence was probably facilitated by reproduction through colony fission.

## Introduction

The monogyne exotic fire ant *Solenopsis invicta* Buren, is one of many species, both plant and animal, that is adapted to exploit ecological disturbance. Communities that develop in such disturbed habitat are unstable and revert to a late stage community whose composition varies with geographic location, soil type and climatic variables. Early succession of such plant communities has been subjected to a great many studies, and general theories of succession have been proposed [1]. Not surprisingly, the communities of insects associated with these plants also change as succession proceeds (e.g.[2]–[5]). Considering the profound changes of the physical and biotic habitat that are usually associated with plant succession, it is perhaps not surprising that ant communities also undergo changes of species composition as succession proceeds. For example, ant species characteristic of early successional stages of reclaimed bauxite mines disappear with time while those of later stages appear [6]. Fire ants decline in abundance as time since habitat disturbance increases [7]. The profound differences in the composition of ant communities in natural and human-disturbed habitats imply that many of the ant species found in human-disturbed habitat would decline if succession were allowed to proceed (e.g. [8], [9].

The fire ant, *S. invicta* is a globally distributed exotic species that has been intensively studied for decades without much attention to how variation in habitat may affect its local and regional distribution and abundance, and thus its impacts [10]–[14]. We should care about both the persistence and relative abundance of fire ants in different ecosystems and for different levels of anthropogenic disturbance because such knowledge will help us understand the ecology and long-term impacts of this exotic species. Recently, long-term observational studies [15], careful habitat gradient studies [16]–[17], and large-scale addition and removal experiments [18]–[21] conducted in the southern USA have provided a better understanding of the local distribution of fire ants in relation to natural and man-made ecosystems and their impacts on co-occurring arthropod populations.

Fire ants are, by far, most abundant in the most altered ecosystems, especially pastures and roadsides [22]–[23]. Heavily disturbed sites in Texas harbored a much higher abundance of *S. invicta*, and *S. invicta* was one of only two ants species found under complete ecosystem alteration [24]–[25]. In the deciduous forests of South Carolina, *S. invicta* appeared in clear-cuts (but not uncut controls) within as little as 3 months post-cutting [26]. *S. invicta* was the most abundant species in highly disrupted habitats such as sugar cane fields [10], lawns [27], roadsides [28], [29], [22], pastures and old fields [30]–[33] and powerline rights of way [34]. In Florida pine flatwoods, a habitat naturally free of fire ants, experiments combining fire ant additions with habitat alteration (mowing and plowing) of intact habitats revealed that establishment and growth of fire ant populations, occurred naturally (without experimental supplementation) primarily in plowed sites [19]. In short, in situations in which disturbed and undisturbed habitats are juxtaposed, *S. invicta* is sharply limited to the disturbed portion [19], [35].

The correlative and experimental evidence reviewed above shows that ecological disturbance is the most likely *cause* of the association of *S. invicta* with early succession habitat. This association is apparent throughout the introduced range, as well as in its native range in South America [36]. However, anthropogenically disturbed ecosystems are unstable, requiring continuous maintenance and, left alone, revert to some late stage condition through plant community succession. The fate of *S. invicta* during the course of such ecological succession has rarely been investigated. Among the few such investigations, Pass [37] found that *S. invicta* was essentially absent once crown closure in pine plantations exceeded 40%. King and Tschinkel [19] showed that mature colonies transplanted to native habitat (in which they do not occur on their own) can persist for considerable time, but their longer-term fate (>3 yr) is unknown. LeBrun et al. [7] report a negative relationship between time since disturbance and fire ant density. Here we report on the fates of *S. invicta* colonies transplanted into native habitats, and of populations self-founded in disturbed habitat after more than 25 years of ecological succession.

## Materials and Methods

### Ethics statement

This study was carried out in the Apalachicola National Forest under US Forest Service permits APA583 and APA56302. No protected species were involved.

Two previous studies served as the starting point for our study— Tschinkel [35] and King and Tschinkel [19]. In 1985, Tschinkel [35] surveyed two species of fire ants (*Solenopsis invicta, S. geminata*) in the coastal plain longleaf pine forests of the Apalachicola National Forest (ANF) south of Tallahassee, Florida. Of the areas he surveyed, the most relevant to the present study was a young longleaf pine plantation in ANF compartment 228 that hosted a substantial population of *S. invicta* as a result of the soil disturbance that occurred during the establishment of the plantation. Using Tschinkel's published site map and a satellite image for the Hilliardville, FL, DOQQ 5136 (Florida Land Boundary Information System: www.labins.org/) the original transects were run again in March, 2012, more than 25 years later, after the site had largely recovered from the original disturbance. Colonies of *S. invicta* were mapped using a Trimble GeoExplorer (CE series) GPS. The data were differentially corrected using the base station maintained by the Florida Department of Environmental Protection, and displayed on the satellite image. The site was also searched more generally for *S. invicta* colonies. As a control, a 750 m section of the closest unpaved forest road that had been surveyed for *S. invicta* in 1985 was resurveyed in June, 2013.

The study by King and Tschinkel [19] reported a fully-factorial experiment in which the native ground cover of 40×40 m plots in the Apalachicola National Forest was mowed, plowed or left undisturbed, and these treatments were crossed with the addition of mature fire ant colonies planted into shallow holes, the addition of soil (without ants) into shallow holes or no additions. This yielded 9 treatments that were replicated at 5 different sites. This experiment addressed the effect of habitat disturbance and fire ants, separately and together, on the native ant fauna. During the study, plots scheduled for fire ant addition were visually surveyed for live colonies twice a year. If the number was too low for the purposes of the experiment, more colonies were added during the cool season. Colonies to be added were collected from roadsides under conditions that favored capturing the queen, that is, cool, sunny mornings. Only queenright colonies could be expected to persist even under the best of circumstances. Experience has shown that about half of the added colonies were probably queenright. The last additions were made early in 2006, and live colonies in these addition plots were surveyed and mapped in the summer of 2006.

In 2012, more than six years after the last additions, teams of four observers visually searched all of the plots for fire ant colonies, this time mapping all colonies in all plots using the GeoExplorer (CE series) GPS, as above. The object was to determine whether the elevated populations of the addition plots persisted in 2012. These data were added to the satellite images along with the approximate locations (estimated from sketch maps) of the 2006 colonies. For both surveys we focused on counting colonies, rather than using pitfall traps or baits, as mature fire ant colonies are conspicuous, and thus accurately counted within the plots [19], [35].

## Results

**Figure 1:**
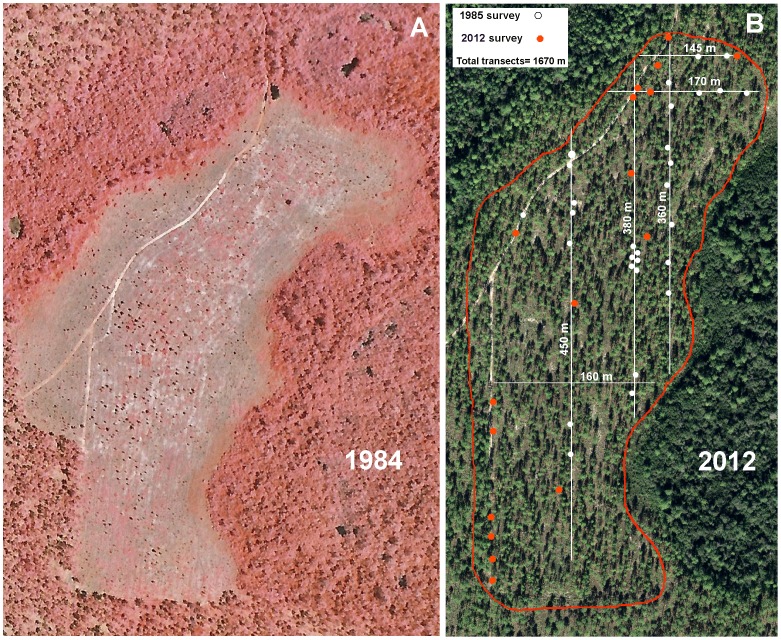
A flatwoods longleaf pine plantation surveyed in 1984 (left panel, false color) and again in 2012 (right panel, true color), showing *S. invicta* colonies detected in 1984 (white symbols) and 2012 (red symbols). Note the furrowed soil and small size of the trees in 1984. White lines indicate transects surveyed both in 1984 and 2012, and the red line shows the boundaries of the12: Aerial photo purchased from USDA Forest Service. Right panel: satellite image from the Land Boundary Information System (www.labins.org/), Hilliardville, FL, True Color DOQQ 5136. Conditions for use are as follows: “Since this data was developed and collected with U.S. Government and the State of Florida funding, no proprietary rights may be attached to it, nor may it be sold to the U.S. Government or the Florida State Government as part of any procurement of products or services.”

The pine plantation in ANF compartment 228 had been naturally colonized by *S. invicta* in the mid 1970s following the soil disturbance associated with establishing the plantation (Fig. 1a). But had succession in the intervening 27 years changed this fire ant population? Determination of such effects requires a comparison with similar populations whose habitat has remained unchanged over this period. A survey of a nearby section of unpaved, occasionally-graded roadside served this purpose. In 1985, this section of forest road harbored 27 fire ant colonies in 730 m of roadside, or 37 per km. In the 2012, this same section contained 29 colonies, or 40 per km. Similar roadsides within 5–10 km averaged about 40–50 colonies per km in both 1985 and 2012. These roadside surveys therefore suggest that in the absence of succession, no change in the population density of fire ants was to be expected.

At the time of the 1985 survey of the pine plantation [35], soil disturbance was still apparent (Fig. 1a), and 29 colonies of *S. invicta* were found along 1.6 km of transects, a density of about 20 colonies per km of transect (Fig. 1b). By 2012, 27 years after the original survey, the ground cover had mostly reestablished itself and little soil disturbance was apparent. A resurvey of the same transects detected only 10 colonies (6.3 colonies per km; Fig. 1b), a significant decline (Chi-square = 6.49, d.f. = 1, *P*<0.01). Of the 29 colonies present in 1985, 26 were not associated with vehicle tracks, whereas in 2012, 5 of the 10 colonies were not thus associated. The decline of colonies not associated with vehicle tracks was thus even greater (Chi-square = 10.66, d.f. = 1, P<0.001).

During the intervening 27 years, the number of colonies associated with vehicle tracks changed little (3 vs. 5). A survey of a longer section of vehicle track not surveyed by Tschinkel (1988) detected another 6 colonies (Fig. 1b, lower left), indicating that *S. invicta* has a strong preference for the disturbance associated with such tracks and populations can persist there for decades.

### Experimental Plots

**Figure 2:**
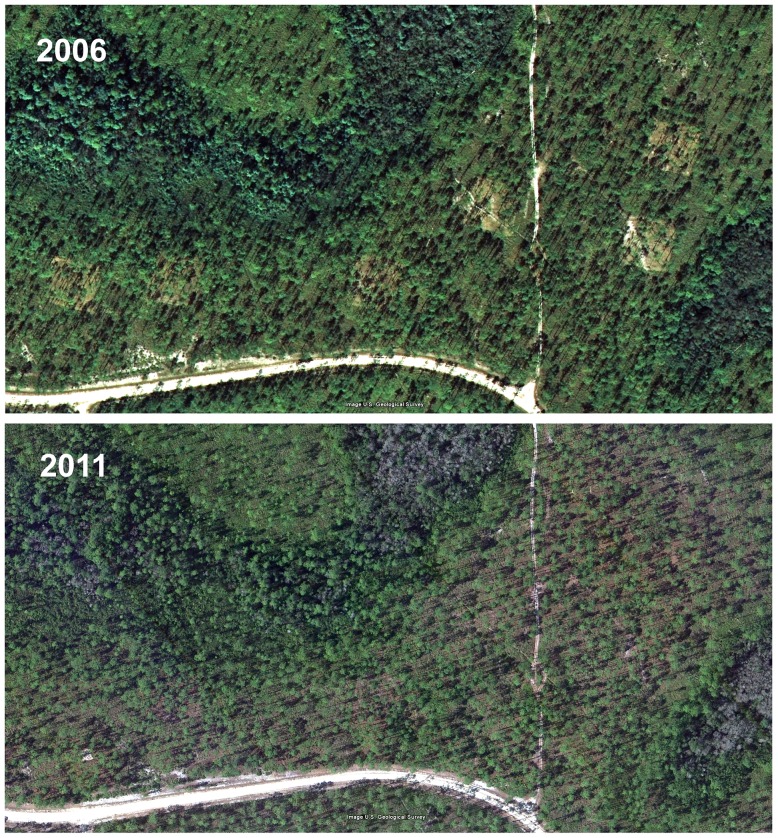
Satellite views modified from U.S. Geological Survey images of a representative of the five experimental sites in 2006 (upper panel) and in 2011 (lower panel). The plowed and mowed 40×40 m plots are clearly visible as pale squares in 2006, but not in 2011, indicating that most of the effects of disturbance had disappeared. On the ground, some effects of plowing were still visible in some plots in 2012, but most mowed plots were no longer distinguishable from the surrounding ground cover. Undisturbed controls are not visible in either year. For conditions for use, see Fig. 1.

Fig. 2 shows the changes in appearance of a representative of one of the five experimental sites between 2006, the last year of disturbance treatments, and 2011, a year before the final survey. In 2006, the plowed and mowed plots are clearly visible as pale squares (Fig. 2, upper panel), but by 2011 these disturbances were no longer visible (Fig. 2, lower panel). On the ground, some of the effects of plowing were still discernible in some of the plots in 2012, but most of the mowed plots were no longer distinguishable from the general ground cover. Control plots are not visible in either year because ground cover was not disturbed.

From 2003 to 2006, fire ant abundance in the King and Tschinkel study [19] was assessed annually with pitfall traps and twice-annual mound counts of addition plots. The 2006 condition of the plots provided the starting point for the comparisons in this paper. The analysis below takes into account that about half of the added mature colonies were probably queenless and did not survive long. Only queenright colonies have prospects of longer survival. In 2006, only the addition plots (n = 15) were visually searched for *S. invicta* mounds, but in 2012, all of the plots were searched (n = 45) to verify the typical density of fire ants throughout the entire area under study.

Adding fire ant colonies to plots greatly increased their abundance. In 2006, the last year of the study, addition plots hosted a mean of 10.6 colonies (95% CI: 8.15–13.1), and had a mean of 24 *S. invicta* per pitfall, whereas the non-addition plots averaged 1.5 *S. invicta* per pitfall (ANOVA, addition treatments: *F*_2,1611_ = 630.2; *P*<0.0000001; Table 1; for details, see King and Tschinkel [19]). This 16-fold difference in trap catch suggests a parallel difference in mound counts (although these were not counted in 2006).

**Table 1:** ANOVA for number of *S. invicta* in pitfalls in 2006 in relation to habitat manipulation and addition of fire ant colonies.

Factor	SS	d.f.	MS	F	p
**Intercept**	128587	1	128587	912	<0.000001
**Disturbance**	1265	2	632	4.49	<0.02
**Fire ants**	177687	2	88843	630	<0.000001
Disturbance*Fire ants	2230	4	557	3.96	<0.004
**Error**	227092	1611	141		

The significant interaction term is the result of higher fire ant numbers in non-addition plowed plots.

By 2012, six years later, the mound count in the addition plots had decreased 74% (s.d. = 26%), from 10.6 to only 2.3 colonies (95% CI: 0.92–3.8) (*t*-test by year, *t*-value  = 6.27; d.f. = 28; *P*<0.0001; Table 2). No pitfall trapping was carried out in 2012, but in light of the decrease in mound counts, a large decrease in pitfall catch would have occurred.

**Table 2:** Mound counts in the “addition plots” in 2006 and 2012.

compartment	plot	treatment	total count, 2006	total count, 2012	change, 2006–2012	% change, 2006–2012
231	1	control	13	1	−12	−92%
232	1	control	14	7	−7	−50%
245-13	1	control	9	0	−9	−100%
245-50	1	control	3	2	−1	−33%
246	1	control	4	2	−2	−50%
231	3	mow	21	0	−21	−100%
232	3	mow	8	2	−6	−75%
245-13	3	mow	7	1	−6	−86%
245-50	3	mow	14	1	−13	−93%
246	3	mow	10	1	−9	−90%
231	6	plow	10	1	−9	−90%
232	6	plow	10	9	−1	−10%
245-13	6	plow	10	1	−9	−90%
245-50	6	plow	13	4	−9	−69%
246	6	plow	13	3	−10	−77%

Mounds were not counted in “non-addition” plots in 2006.

Mound counts for 2006 can be approximated indirectly from pitfall captures. In 2012 there was no significant difference in mound numbers among any of the treatments, addition or non-addition (ANOVA), which means that by 2012 the addition plots had declined to the same level as the non-addition plots (mean 2012 non-addition = 1.61 colonies, s.d. = 0.97; one outlier was deleted, more on that below) Non-addition plots consisted of control plots with 0.7 colonies and mow plots with 1.3 colonies. Thus between 1 and 2 colonies per plot can be regarded as the “natural” background density, a density that produced about 1.5 *S. invicta* per pitfall in 2006. Therefore, supplemented fire ant populations returned to background densities, whether measured as mound counts or pitfall catches. For the addition plots in 2012, the background abundance of 1–3 *S. invicta* per pitfall represents a large decrease from about 24 per trap in 2006. These background densities are probably attributable to the pervasive presence of small scale soil disturbance from vehicles, “stump pulls,” “borrow pits” and other forest service and recreational activity in this forest.

**Figure 3:**
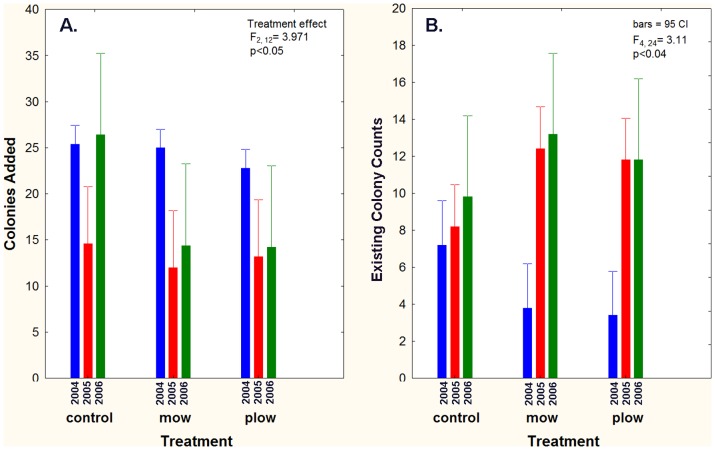
Mature colony transplantations. A. Number of colonies added to treatment plots by year. Initial additions were the same for all plots, but controls required more additions in subsequent years. B. Existing colonies were counted before additions. Persistence of transplanted colonies was higher in the mow and plow plots than in the controls.

The lack of persistence of *S. invicta* at even moderate densities in later successional stage native longleaf pine savanna habitat is emphasized by the fact that colonies had to be added every year in order to maintain the *S. invicta* densities called for in the experiment (Fig. 3). Not surprisingly, more colonies were added in 2004 than the other two years (means = 24, 13, 18 respectively; repeated measures ANOVA, effect of year, *F*_2,24_ = 10.1063, *P*<0.001). Live mounds were counted each year just before the additions were made, and the number added adjusted accordingly (about half the colonies were probably queenright and had no prospects of surviving long, see Methods). Through the initial addition of 24 colonies per plot in 2004, mean colony counts increased from 4.8 to 10.8 in 2005 (Table 2). Addition of another 13 colonies per plot in 2005 increased the counts to a mean of 11.6 in 2006 (effect of year on counts: repeated measures ANOVA, F_2, 24_ = 23.53, *P*<0.00001). However, when habitat treatments are considered, additions increased the counts in mow and plow plots more than in control plots, so that by 2005 and 2006, mow and plow counts were significantly higher (means 12–13) than controls (means 8–10) (year by treatment interaction, F_4,24_ = 3.112, p<0.04). This was in spite of 15 to 26 colonies being added to control plots in 2005 and 2006, but only 12 to 14 to the mow and plow plots. In other words, losses of transplanted colonies were higher in the control plots than in the disturbed plots, requiring more annual additions, but even then not achieving the same colony counts in controls as in the other two treatments. This suggests that undisturbed native habitat is less congenial to fire ant persistence than disturbed habitat.

Projecting forward to 2012, even the three repetitions of plowing did not alter the habitat enough to allow *S. invicta* colonies to persist for 6 years, so whatever it was about disturbance that improved year-to-year persistence had disappeared within 6 years.

### An outlier

**Figure 4:**
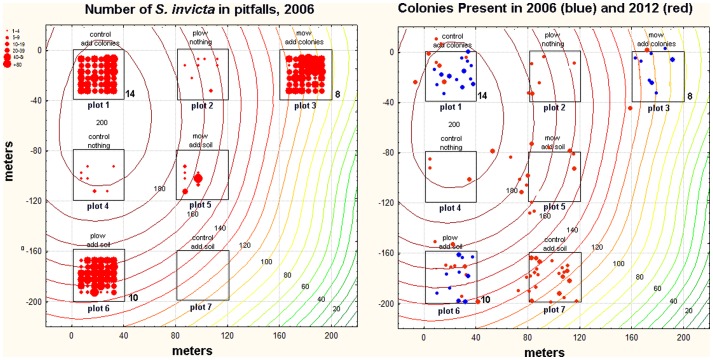
Presence of *S. invicta* in compartment 232, plots 1–7. Contour lines show the elevation (cm) above the wetlands to the lower right and bottom. Boldface numbers next to plots 1, 3 and 6 are the number of fire ant mounds present in 2006. Left panel: *S. invicta* abundance in pitfalls in 2006. The size of the symbol indicates the number captured (legend at upper left). Right panel: mounds detected by survey in 2006 (blue symbols) and 2012 (red symbols). Although only “add colonies” plots were surveyed for mounds in 2006, the low 2006 capture rates in pitfalls of “non-addition” plots suggests that few mounds were present in those plots. Survey of plots 6 and 7 in 2013 found most of the colonies to be polygyne.

Plot 7 in compartment 232 was an interesting exception to the general pattern. It was a control-add soil treatment in which no *S. invicta* were detected in pitfalls in 2006 (Fig. 4, left). However, when surveyed for mounds in 2012, it contained 26 mounds (Fig. 4, right). On a sunny, cool morning in February 2013, 18 mounds in and near plot 7 were checked for queen number by scattering mound soil in large photo trays and inspecting for queens. Of the 18 mounds checked, 11 were confirmed as polygyne by the detection of 2 to 5 queens that were both physogastric and attractive to workers. Of the remaining 7 nests, a single queen was found in 2 and no queen in 5. However, almost all nests, whether queens were seen or not, had the small, pale workers and absence of sexual brood that is characteristic of polygyne colonies, and were probably polygyne. Polygyny was probably wide-spread in this site in general, as several mounds were present in other non-addition plots (Fig. 4, right).

Polygyne colonies were unknown in the Tallahassee region until recently, but since their arrival, they have been spread by the county road department during road maintenance [38]. Polygyne colonies are not known to reproduce successfully through mating flights and independent founding [39]. It is possible that some of the colonies we added were polygyne, although it seems unlikely given the complete absence of fire ants in 2006 pitfalls and colony surveys. Whether they will persist for long is an interesting question.

## Discussion

Our data show that monogyne *S. invicta* can persist in this native habitat at only low levels and often in association with small-scale soil disturbance. About half of the non-persistence from year-to-year was probably because half of the transplanted nests in the previous study were queenless [19]. However, even considering that approximately only half the colonies were queenright, persistence was much less than 100%. Thus, over the three years, 65 colonies were added to the control plots, of which perhaps 30 were queenright, but by 2006, only 10 colonies (one-third of queenright) were present. Similarly, about 50 colonies were added to the mow and plow plots. Assuming about 25 were queenright, only 12 to 13 persisted in 2006, a survival rate of about 50%. However this was a better survival rate than resulted in King and Tschinkel's [20] study. They added 234 colonies to experimental plots from 2004-6 in a longleaf pine savanna ecosystem on Brickyard soil (a perched clay layer, as opposed to the pure sandy spodosols of this study) and only 66 colonies were found in the addition plots at the end of the study. In King and Tschinkel's [20] study, fire ants were already present in higher densities (6 colonies, on average, per 40×40 m plot in control plots). This suggests a survival rate of around 15% for added colonies, much lower than the present study, which is likely explained by the poor habitat (for ants) in the King and Tschinkel [20] study.

The non-persistence that is a common theme in all of these studies may in part have been due to the difficulties of establishing a new nest and territory in the new environment, leading directly to colony death. Of colonies that established successfully and persisted for at least a year, longer-term non-persistence was most likely the result of a lack of recruitment— planted colonies probably lived out their life span (average 7–8 years, [40], [41], but after they died were not replaced by newly founded colonies. Many of the planted colonies will not have been in the bloom of youth, so that colony mortality was probably continuous during the six year interval. The age of transplanted colonies was unknown, but assuming they were sampled from a stable population, probably averaged 3–4 yr with a right-skewed age distribution [23].

The fact that mature colonies experimentally transplanted to undisturbed habitat in these same forests can persist for more than a year, but mostly vanish within 6 years suggests that the road block to persistence is associated with colony reproduction, i.e. recruitment. Recruitment itself can be dissociated into the habitats that mated queens choose to settle in after mating and their success in founding a colony in the chosen habitat. Newly mated *S. invicta* queens show a strong preference for disturbed habitat and fail to found successfully in native habitat (King and Tschinkel, unpublished data). That is, queens choose to settle in habitat in which they can successfully found new colonies. Just what habitat characteristics produce this difference is unknown, but should be the focus of future studies because the combination of preference and success is a fundamental process in the assembly and evolution of communities. Morrison [15] observed a decline in fire ant densities, possibly related to succession and Morrison and Porter [42] observed a positive association between fire ant densities and generalized ant and arthropod diversity further suggesting that habitat quality is a key feature pf early successional habitats for many insect species.

In a sense, the persistence and possibly novel appearance of polygyne *S. invicta* in compartment 232 supports the hypothesis that the observed failure of colony founding by newly mated monogyne queens in native habitat is a major barrier to persistence. Because polygyne *S. invicta* reproduce by colony fission, they avoid this bottleneck and persist. How long they can persist is an open question. Even the massive polygyne invasion described by Porter and Savingano [43] had largely abated within 10 years [15].

The habitat disturbance caused by mowing and plowing faded over the six elapsed years, although the effects of plowing on the ground cover were still visible. Colony-founding queens of *S. invicta* are attracted to disturbed habitats (unpublished data), but are apparently either unable to establish colonies even in the plowed plots, or the colonies did not survive for 6 years. Whatever the cause, it is clear that even though *S. invicta* colonies are abundant along graded forest roads, they rarely establish and/or persist within the adjacent native habitat. Even those that do persist within the native ground cover are often (but not always) associated with small disturbances that have exposed bare soil (unpublished observations). Human incursions into this habitat are many— hunters, off-road vehicles, old logging scars, old drainage ditches and so on.

Longer-term, successional decline of fire ant populations similar to our pine plantation study has also been reported by LeBrun et al. [7] whose south Texas study showed in a chronosequence that fire ant abundance decreased with the time since disturbance. Similarly, ant species typical of early succession declined in reclaimed bauxite mine sites as they aged and revegetated [6]. The fact that early succession habitats are populated by distinct communities of ants that are different than those of late successional stages [6], [8], [9] suggests that if succession were allowed to take place, many of the ant species in the early-stage communities would decline or disappear.

Studies of unusual and persistent occurrences of fire ants populations in a few types of intact ecosystems lacking anthropogenic disturbance provide further insight into the attributes of altered habitats that favor fire ants. Specifically, moist habitats with high water tables, little or no canopy, clay or loamy soils, and frequent natural disturbances such as seasonal flooding, and fires [23], [35], [44] sometimes support fire ant populations averaging approximately 1/6 the densities of pastures and roadsides in the same region [17], [20], [40]. Wetland margins, prairies, and frequently flooded areas within some longleaf pine forests are the most common examples [17], [20]. Such natural habitat disturbances may act as barriers to most native species while simultaneously favoring fire ants [20], and may be somehow quite similar to the habitat features created by anthropogenic disturbances. Additional support for this comes from Stuble et al. [21] who treated plots in native habitat that had substantial densities of both fire ants and native ants with poison baits, reducing all ant densities. Fire ants did not preferentially recolonize these treated plots.

The association of *S. invicta* with mostly human-caused disturbance has been amply demonstrated [23]. The focus now needs to shift to the question of just what life history characteristics are matched to the characteristics of disturbed habitats, and what behavioral and physiological attributes of the ant allow it to seek out, colonize and persist in such habitats. We have shown that populations self-founded in disturbed Florida coastal plain pine forest dwindle as the habitat recovers from the disturbance and reverts to a condition approximating the original undisturbed state. This suggests that certain characteristics of the late-succession habitat are not congenial to monogyne *S. invicta* survival or recruitment, or both. The transition from congenial to not congenial can be as narrow as a few meters between the edge of a maintained forest dirt road and the adjoining native ground cover.
